# Application of an innovative grid-based surveillance strategy to ensure elimination and prevent reintroduction of malaria in high-risk border communities in China

**DOI:** 10.1186/s12889-022-13753-1

**Published:** 2022-07-14

**Authors:** Shen-ning Lu, Wei Ding, Jia-zhi Wang, Shou-qin Yin, Sheng-guo Li, Xing-wu Zhou, Qiu-li Xu, Xiao-dong Sun, Chris Cotter, Michelle S Hsiang, Allison Tatarsky, Roly Gosling, Shan Lv, Duo-quan Wang

**Affiliations:** 1 Chinese Center for Disease Control and Prevention (Chinese Center for Tropical Diseases Research), NHC Key Laboratory of Parasite and Vector Biology, National Institute of Parasitic Diseases, WHO Collaborating Centre for Tropical Diseases, National Center for International Research On Tropical Diseases, Shanghai, 200025 China; 2 Tengchong Center for Disease Control and Prevention, Tengchong, China; 3 Yunnan Provincial Research Center of Arbovirus, Yunnan Institute of Parasitic Diseases, Yunnan Provincial Research Center of Malaria, Pu’er, China; 4 Pudong New Area Center for Disease Control and Prevention & Pudong Institute of Preventive Medicine, Fudan University, Pudong New Area, Shanghai, China; 5 Malaria Elimination Initiative, Institute for Global Health Sciences, University of California San Francisco, San Francisco, California USA; 6 Department of Women’s and Children’s Health, International Maternal and Child Health, Uppsala University, Uppsala, Sweden; 7 Department of Epidemiology and Biostatistics, School of Medicine, University of California San Francisco, San Francisco, USA California; 8 Department of Pediatrics, University of California San Francisco, Benioff Children’s Hospital, California San Francisco, USA; 9 Department of Control of Disease, London School of Hygiene and Tropical Medicine, London, United Kingdom; 10 School of Global Health, Chinese Center for Tropical Diseases Research, Shanghai Jiao Tong University School of Medicine, Shanghai, 200025 China

**Keywords:** Malaria elimination, Grid-based strategy, Mobile and migrant populations, China–Myanmar border region

## Abstract

Grid management is a grassroots governance strategy widely implemented in China since 2004 to improve the government’s efficiency to actively find and solve problems among populated regions. A grid-based strategy surveillancing high-risk groups, including mobile and migrant populations (MMPs), in the China–Myanmar border region has played an indispensable role in promoting and consolidating the malaria elimination efforts by tracking and timely identification of potential importation or re-establishment of malaria among MMPs. A sequential mixed methods was implementated to explore the operational mechanism and best practices of the grid-based strategy including through the focus group discussions (FGDs), comparison of before and after the implementation of a grid-based strategy in the field sites, and data collection from the local health system.This paper distills the implementation mechanism and highlights the role of the grid-based strategy in the elimination and prevention of re-establishment of malaria transmission.

## Introduction

The World Health Organization (WHO) awarded China malaria-free certification on June 30, 2021[1]. Despite this achievement, Yunnan Province, located in southwestern China, faces the risk of malaria resurgence threatening China’s efforts to prevent the re-establishment of malaria [2]. The geography of Yunnan Province includes lowlands and steep altitudes up to 6,740 m, and enjoys a climate ranging from tropical to subzero with varying suitability for mosquito breeding. Yunnan Province belongs to the Greater Mekong Subregion (GMS), which shares a 4,060 km long porous, natural, barrierless border with malaria-endemic countries including Myanmar, Laos and Vietnam. Approximately 30% of the nearly 3,000 imported cases in China every year are reported from the China-Myanmar border region largely due to frequent local population movement. A total of 2,219 imported malaria cases were reported in Yunnan Province between 2014–2019, accounting for 97.2% of the total reported cases in the province[3]. The potential spread of artemisinin-resistant *Plasmodium falciparum* threatens malaria elimination and prevention of re-establishment efforts in this border region. Furthermore, poor access to local health services also poses significant risks. The Yunnan border region is the most underdeveloped area within China, and the residents living in these marginalized regions are at high risk for malaria infection, especially mobile and migrant populations (MMPs) who cross the border daily through informal border-crossing points for business, schooling, or medical care[4]. Moreover, active case screenings conducted by the local malaria program in the community may be unable to track MMPs who are likely to travel back from high-transmission areas. Therefore, it is important to improve access to malaria screening, diagnosis, treatment and prevention for MMPs, in order, to prevent re-establishment of malaria in Yunnan border areas.

A high-performing health system to ensure early detection, notification and prompt treatment is identified as one of five key points for preventing the re-establishment of malaria according to WHO’s *A Framework for Malaria Elimination *[5]. Therefore, detecting and treating infections in cross-border MMPs is of great significance to maintain malaria-free status in China, while the traditional health system surveillance and response system may miss these populations. For these reasons the China malaria elimination program has utilised an extension of the government adminsitrative system, the grid system, to try and reach such populations.

The grid system is a grassroots governance strategy that reallocates administrative resources at a neighborhood level and provides necessary trainings to community members, which orginated from the 2003 SARS crisis [6, 7], and is maintained by the Chinese government in case of social crisis. The term “grid” was introduced as the lowest level of urban governance below urban communities, covering a small area of roughly 10 km^2^. A grid administrator, from the community and selected by the local community as part of the broader administrative system, monitors the community members while at the same time provides services to the local community. The grid-based strategy relies on a variety of techniques, such as information sharing, following-up evaluations of people’s satisfaction levels, and liaising with political-legal agencies such as the police, judiciary, and wider administrative systems [8]. The effectiveness of the grid-based strategy has been evaluated during dengue fever outbreaks in urban and rural settings [9], and its value has become more prominent during the COVID-19 pandemic [10]. The grid-based governance has been standardized and enhanced since the COVID-19 outbreak in China, contributing to containment of the virus at the neighborhood level. Besides taking routine body temperature check of residents, checking residents’ travel histories, transferring infected residents to designated hospitals and monitoring quarantined households are also included in the ‘carpet-style’ investigations to track infected cases [7].

The grid-based strategy among MMPs in the China–Myanmar border region has played an indispensable role in promoting malaria elimination efforts. Implemented by multisectors at the grid area under the guild including supervisor of health facilities, residents, family and community that active participate in the action, the grid-based strategy supports the tracking and timely identification of any potential importation or re-establishement of malaria among MMPs in the border region. This paper aims to distill the operational mechanism of the grid-based strategy for malaria elimination in the China–Myanmar border region, and emphasizes the role of the grid-based strategy in elimination and preventing the reestablishment of transmission.

## Methods

### Study design

The study design contains a sequential QUAL-QUAN mixed methods study with a descriptive quanlitative followed by a quantitative component.

### Study setting

Yunnan Province—situated in the southwestern part of China with an area of 394,000 km^2^ and 4,061 km of border with Myanmar, Laos and Vietnam— has a population of 45 million [11]. Historically, Yunnan Province has stable malaria endemicity due to its mountainous valleys, proximity to the Indian Ocean and a Pacific monsoon climate along with frequent human movement contributing to the highest burden of malaria in the areas that border Myanmar. The border areas face many challenges including continuous importation of malaria infections, increases in population movement and wide distribution of efficient vectors [11]. Tengchong County, located at the westernmost edge of Yunnan Province, was chosen for the quantitative analysis because it has the highest malaria incidence in  the China–Myanmar border region [12].

### Study population

For the qualitative study, the study population included key informants who possessed experiences of relevance to the grid-based strategy, including the program managers, community health workers, medical providers, and community leaders working or living in the catchment area for this study. A total of 36 individuals including 4 participants from county-level centers for disease control (CDC) and 32 participants from 16 township hospitals (2 from each sample hospital) were interviewed through focus group discussions (FGDs) to assess the knowledge and practices of malaria case tracking, case reporting, case investigation, and reactive case detection (RACD) through the grid-based strategy. For the quantitative study, data on risk population, malaria case reporting, case investigation, and RACD from Tengchong County were extracted from the patients registered in local CDC and 16 township hospitals for the 7-year period from January 1 2013 to December 31 2020.

### Sources of data and data collection

The implementation of the *China Malaria Elimination Action Plan (2010–2020)* in the Yunnan border area and the adaptation that took place with the grid-based approach were explored through FGDs with local participants. A team of three researchers conducted the FDGs after obtaining written informed consent. Participants were informed the purpose before the data collection. FGDs lasted for about 90 to 120 minutes. A pilot-tested FGDs guide with broad open-ended questions was developed prior to the group discussions with local key informants, in oder to identify key questions to be covered as well as to ensure the cultural adaptability, reliability, and validity of the study design and questions. Four FGDs were conducted with 6–8 participants (a total of 36 participants) present in each FGD. The FGDs were audio-recorded and notes were written by researchers during data collection. Interviews were carried out between June 6 -30 2021.

Data were extracted from the patients registered in local CDC and 16 township hospitals for the 7-year period from 1 January 2013 to 31 December 2020. The data variables included MMPs, at-risk population, malaria case reporting, case investigation, and RACD.

### Data analysis

Audio-recorded tapes from FGDs were manually transcribed and translated into English by the FGDs facilitators. The translated transcripts were analyzed using qualitative data analysis software (QSR NVivo 11). And quantitative data were double entered into Microsoft visual FoxPro (version 9.0) and transferred to STATA (version 9.2) for analysis. All data were summarized and verified with the township hospitals and local CDC within 15 days after completion of field survey.

### Ethical consideration

This study has been approved by the Ethical Review Committee of Chinese Center for Disease Control and Prevention. Written informed consent was obtained from each respondent for the participation in FGDs.

## Results

### Qualitative results

Two overarching themes emerged from FGDs relating to: (a) operational mechanism of the grid-based surveillance strategy; and (b) adaptation of the grid-based surveillance strategy in practice.

### Theme 1: Operational mechanism of the grid-based strategy for malaria surveillance: a case in Tengchong.

#### Stucture of grid-based surveillance in MMPs

Tengchong, the border county to Myanmar, has a vertical-horizontal combined structure to monitor MMPs.^1^ In term of vertical structure, the MMPs’ information was reported from the village to township to county and to provincial levels then up to the national level to the Migrant Population Service Center (MPSC), an affiliation to the National Health Commission, through the annual China Migrants Dynamic Survey (CMDS) program (https://www.chinaldrk.org.cn/). As requested by the CMDS program MMPs' information should be collected including: (1) family members and family income/expenditure; (2) employment status; (3) basic public health and family planning services, and; (4) medical and health services for the elderly [13]. The horizontal structure characterized by the grid-based strategy across the communities supports the annual national MMPs survey as well as day-to-day MMPs surveillance. This grid-based strategy has been implemented for malaria community case management since 2016 [14].

### Application of grid-based approach for malaria survelliance (Fig. 1)

**Fig. 1:**
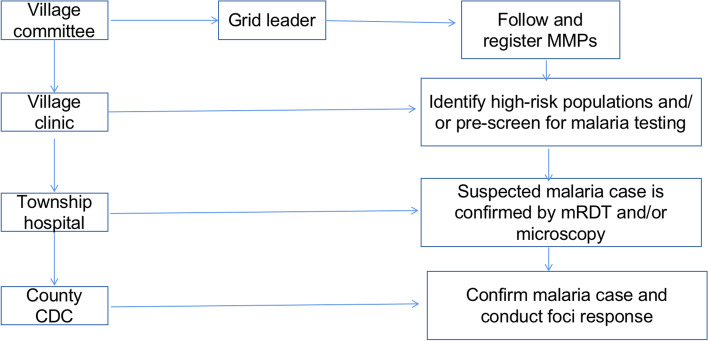
Flowchart of the grid-based strategy for malaria case management in MMPs in Tengchong County, China–Myanmar border region. Note: MMPs, mobile and migrant populations; mRDTs, malaria rapid diagnostic tests; CDC, center for disease control and prevention

Major functions of grid-based management are: effectively managing the MMPs; identifying people at risk of malaria infection; taking interventions to reduce the risk of malaria infection; timely detection of malaria cases. These functions are achieved by institutions within a grid such as community groups, village committee and village clinic.

In Tengchong, key members of a grid unit to conduct malaria case surveillance include grid administrator, village committee staff and village doctor. The grid administrator selected by or volunteered from the local community is the leader of the community groups. The grid administrator collects the necessary information (i.e. travel plan/history) on MMPs within the community and submits the information to officers at the point-of-entry, and a copy to the local village committee and village doctor via a mobile messaging application. The village committee staff is responsible for checking and registering the information. In some villages, for example, the village committee convenes monthly meetings with the village doctor to confirm the information of MMPs. The information from the grid administrator and the village committee staff are not the only sources of information for the village doctors. The village doctor also registers the MMPs missed by the formal registration process during healthcare delivery. With this information, the village doctor develops a detailed spreadsheet with MMP departure and arrival information including the date and duration of travel, point-of-entry/exit, travel destination, and companion information.

Based on above information, the village doctor can identify high-risk populations^2^ and deliver targeted services, such as health education on malaria prevention, deliver long lasting insecticide-treated bednets and other preventive tools for those who are ready to depart, refer febrile and suspected patients to higher-level medical facilities, follow-up case treatment, and assist the local county Centers for Disease Control and Prevention (CDCs) on foci investigation and clearance, and supervise implementation of the spring treatmemt.

### Theme 2: Adaptation of the grid-based surveillance strategy in practice

According to the National Malaria Elimination Action Plan (2010–2020), township-level health facilities are the lowest units that diagnose, treat, and follow up malaria cases in China’s malaria elimination program structure. Yet, according to the results of FGDs, when malaria incidence was high in these border areas, community surveillance, case management, and response activities were shifted to the grid strategy engaging the village doctors, village committees, and community groups. Table 1 shows the different practices before and after the implementation of grid-based surveillance. This strategy remains in place through the successful elimination of malaria and ongoing prevention of re-establishment.

**Table 1:** Different practices for community case management of malaria before and after the implementation of grid-based strategy in Tengchong County, China–Myanmar border region

Activities	Before grid-based strategy	After grid-based strategy
Health education and promotion for local residents	Conducted by township hospital	Conducted by the grid members including village doctors, village administrators, village committee leaders and security workers of village/community
Distribution of malaria preventive package for MMPs before they depart	Conducted by township hospital	Conducted by the village doctors of the grid area
Registration of MMPs before leaving and after returning	Not conducted	Family report to the grid’s workers who will make registration
Malaria symptom surveillance among local residents and MMPs	Not conducted	After family report, village doctors will measure temperature and then report the case through computer
Medical treatment seeking of residents or MMPs with fever	Patients sought for treatment individually	Village doctors register and report, test the patients using malaria RDTs in key and remote area, then guide patient to hospital
Malaria diagnosis	Conducted by the township hospitals or county hospitals	Conducted using RDTs which are provided in some key or remote grid areas. Patients will be guided to township or county hospitals for further diagnosis
Malaria treatment	Treatments were given by the county CDCs with following-up through telephone	Conducted by the township hospitals with follow up by village doctors or other grid members
Training on malaria control	Township-level trainings were provided by the county level, with few trainings at the village level	Township-level trainings were provided by county level; village-level trainings at were provided by the township level

Note: *MMPs* mobile and migrant populations, *CDCs* centers for disease control and prevention, *RDTs *rapid diagnostic tests

### Quantitative results

The application of the grid-based strategy allows for a more targeted proactive surveillance approach based on known high-risk populations. The implementation of a grid-based strategy has increased the screening positivity rate, which rose from 1.04–2.71% (through universal screening) in 2013–2015 to 31.8–56.14% in 2016–2020 by screening the identified (targeted) high-risk populations in these border areas. As a result of early detection and response to imported cases in which the grid-based strategy has played a critical role, there were no local malaria cases for five consecutive years from 2016 to 2020 in Tengchong County, which reported the most imported malaria cases during 2013–2019. Tengchong County became the first county to pass the national evaluation of malaria elimination in 2016 among the 18 counties in this border region, and two years ahead of the original timeline [15].

## Discussion

With the recent stalled global progress in reducing malaria mortality and morbidity, WHO has updated its Global Technical Strategy for Malaria 2016–2030 in 2021, with an emphasis on providing the appropriate interventions to the populations that need them, instead of a one-size-fit-all approach [16]. The grid-based surveillance strategy is a useful approach for countries at its last mile of malaria elimination or countries that have already achieved malaria-free status to prevent the re-establishment of local transmission.

There are three main benefits of the grid-based management: (1) leveraging the proactivity of the basic units in social governance system, (2) creating rapid interaction between residents and government, and (3) integrating available resources through multi-sectoral collaboration [17]. In our case, the grid-based surveillance strategy increases the effectiveness of community case management of malaria (CCMm) with a proactive approach based on the targeted high-risk populations. It allows information to be collected in a more comprehensive way on MMPs’ basic conditions and travel history, and through regular field visits and communication with the local community, greater communication and mutual understanding among local key stakeholders is strengthened to accelerate malaria elimination efforts. By emphasizing the multi-sectoral approach, it promotes information sharing and breaks down communication barriers among key stakeholders, including the local public security department, the local health department, and the local entry-exit inspection, and quarantine department, the grid-based strategy facilitates rapid response to the confirmed cases and to the foci to prevent potential onward transmission. It gives an example of increasing local stakeholders’ ownnership in the delivery of malaria control and elimination activities and how community organizations can link to health systems to improve public health impact and to maximize resources from all sectors to solve malaria delivery strategies and develop tailored and targeted approaches suitable to local context.

In global malaria control, multi-stakeholder involvement and community engagement are quoted as vital for long-term cost effective success in malaria control and eliminationyet are frequently overlooked in programming. The grid-based strategy provides a solution of how to operationalize community engagement [18] and multi-stakeholder involvement [19]. Strengthening lower level health program involvement in the design and iteration of delivery strategies is recognized as the future of malaria control in Africa, where national-led strategies need to be tailored by district-level decision-making to suit local context and populations, providing the district with more flexibility and resources for demand-driven and problem-based solutions to local operational challenges [19]. The grid-based strategy gives an example of how community organizations can link to health systems to improve public health impact and to maximize resources from all sectors.

The grid-based surveillance strategy did identify some challenges that need to be considered further. First, beyond the MMPs who do legal activities, there is a need to determine how to reach and work with more illegal or hidden populations who could benefit from health services in the region. Second, a lack of standardized work procedures and quality of care provided by different grids can be variable. The UNICEF M&E indicators for monitoring community level initiatives might be introduced towards a more standardized strategy to measure effectiveness across different grids. Third, the grid administrators are volunteers and are not receiving compensation. There is a need to develop incentives for the extra responsibilities for sustained and stable-quality of service.

## Conclusion

In the context of malaria elimination in the GMS, MMPs who move from their permanent residence to malaria-endemic areas for work or other purposes are a key population threatening elimination targets. Therefore, the WHO has called upon governments in the GMS to include activities targeted at them [20]. Difficulty in managing these high-risk populations can hinder malaria elimination efforts. The successful consolidation of malaria elimination gains in Yunnan Province and China requires effective strategies to be further employed to maintain robust public health infrastructure for disease surveillance. The grid-based surveillance strategy has acted as a cornerstone of community malaria case management in the Yunnan border area, which can reach high-risk populations to accelerate malaria elimination efforts and potentially be scaled-up in similar areas within the GMS. 

## Data Availability

The study datasets are available from the corresponding author on reasonable request. **Ethics approval and consent to participate.** This study has been approved by the Ethical Review Committee of Chinese Center for Disease Control and Prevention. Written informed consent was obtained from each respondent. All methods were carried out in accordance with relevant guidelines and regulations. **Consent for publication.** Not applicable.

## Footnotes

1 The targeted MMPs are people older than 15 years, whose registered permanent residence (also called ‘Hukou’) who do not belong to the locality and have stayed for more than one month in a place.

2 High-risk populations are MMPs who stayed in malaria endemic areas abroad and stayed at least one night in the previous month.'.
